# Nomogram-based risk prediction model employing serum biomarkers to assess intestinal injury risk in patients with metabolic syndrome

**DOI:** 10.3389/fendo.2025.1579833

**Published:** 2025-06-16

**Authors:** Yongqing Chen, Guangxu Wen, Hongmei Wang, Qiyu Yang, Zilang Luo, Xin Wang, Jing Ouyang, Jiadan Yang

**Affiliations:** ^1^ Department of Pharmacy, The First Affiliated Hospital of Chongqing Medical University, Chongqing, China; ^2^ College of Pharmacy, Chongqing Medical University, Chongqing, China; ^3^ Department of Gastrointestinal Surgery, The First Affiliated Hospital of Chongqing Medical University, Chongqing, China; ^4^ Department of Radiation Oncology, Chongqing University Cancer Hospital & Chongqing Cancer Institute & Chongqing Cancer Hospital, Chongqing, China; ^5^ Clinical Research Center, Chongqing Public Health Medical Center, Chongqing, China

**Keywords:** metabolic syndrome, intestinal injury, serum biomarkers, risk prediction model, nomogram

## Abstract

**Objective:**

Patients with metabolic syndrome (MetS) are more likely to have intestinal injury that may accelerate the disease process. We developed a risk prediction model for the non-invasive, rapid, and accurate assessment of intestinal injury in patients with MetS based on serum biomarkers.

**Methods:**

Patients with MetS who underwent colonoscopy were enrolled in this study. Based on the results of the colonoscopy, the participants were divided into the intestinal injury and non-intestinal injury groups. Blood samples were collected to detect laboratory indicators and quantify serum biomarkers. Univariate and multivariate logistic regression analyses were employed to identify predictors of intestinal injury in patients with MetS and to construct a nomogram-based risk prediction model. We employed bootstrapping and 5-fold cross-validation to validate the model internally, with the area under the curve (AUC) used to assess the predictive efficacy, the calibration curve utilized to evaluate the calibration degree, and decision curve analysis (DCA) used to evaluate the clinical practicability of the model.

**Results:**

The study included 263 participants. Our multivariate logistic regression analysis indicated that clinical features such as age, body mass index, neutrophil percentage, as well as serum biomarkers including diamine oxidase and lipopolysaccharide, were predictive factors for intestinal injury in patients with MetS. The model had strong repeatability (bootstrap method: precision: 0.873, 5-fold cross-validation: AUC: 0.948 ± 0.012), differentiation (AUC: 0.957), and accuracy (Hosmer-Lemeshow χ^2^ = 3.985, P = 0.858), while DCA results confirmed the clinical utility of the nomogram.

**Conclusions:**

Serum biomarkers are effective variables to assess intestinal injury in patients with MetS via our nomogram-based risk prediction model.

**Clinical trial registration:**

https://www.chictr.org.cn/, identifier ChiCTR2400088476.

## Introduction

Metabolic syndrome (MetS) is a clustering of medical conditions that include obesity, hyperglycemia, hyperlipemia, and hypertension (1). The MetS mortality rate is much higher than that of any involved disease. MetS is usually accompanied by severe impairment of physical and mental functions. In the United States, MetS is estimated to affect 34.7% of adults (2). It has become a worldwide health issue. In humans, the intestinal mucosa is the first barrier to the external environment and is crucial for preventing the infiltration of harmful pathogens and the absorption of toxins. However, patients with MetS are prone to intestinal damage. Geng et al. reported that the intestinal microecology of obese patients was disturbed, leading to a rise in pathogen levels and a reduction in secreted butyrate, which normally acts as an antioxidant and provides lasting protection for the intestinal mucosa (3). Similar changes have also been observed in the intestinal tract of patients with diabetes (4). These studies suggest that MetS and its component medical conditions can lead to intestinal damage by causing an imbalance of the intestinal microbiota. In addition, MetS and its component diseases can cause intestinal mucosal damage via vascular lesions and microcirculation disorders. Studies have shown that the level of adiponectin in patients with MetS is greatly reduced, and the blood vessels are in a state of oxidative stress, which may eventually lead to vascular damage (5, 6). At the same time, the renin-angiotensin system of patients with MetS is in an activated state that leads to increased angiotensin II levels, vascular inflammation, and atherosclerosis (7). All mentioned manifestations can cause intestinal microcirculation disorders and ultimately lead to injured intestinal mucosa.

When the intestinal mucosa is damaged and microorganisms enter the blood, patients with MetS experience prolonged inflammation, and immune responses ensue, leading to significantly increased cardiovascular disease mortality (8). The relationship between intestinal flora disturbance and intestinal barrier damage, as well as that between chronic inflammation and intestinal barrier damage are mutually causal. This creates a vicious cycle and aggravates intestinal barrier damage (9). Since the intestinal barrier is critically important in the occurrence and progression of MetS, the assessment of intestinal barrier function is important before MetS treatment.

The methods for evaluating intestinal barrier function include intestinal mucosal histological examination, blood indicators, stool, and urine testing. Among these methods, colonoscopy for intestinal mucosal histological examination is the primary means to evaluate intestinal barrier function (10). However, colonoscopy is invasive. It requires preoperative procedures, carries potential safety risks, and demands high patient compliance (11). A study involving 618 patients with Crohn’s disease showed that endoscopy was the least accepted procedure, as it requires bowel cleaning, may cause abdominal discomfort, cost a lot, and might be accompanied by a certain risk of intestinal perforation (12). Serum biomarkers can be easily quantified from blood samples. Compared with colonoscopy, serum biomarker detection offers distinct benefits in terms of being a non-invasive, speedy, simple, and economical choice. Its application in the diagnosis of intestinal injury is more prevalent (13). Some studies have confirmed the feasibility of certain serum biomarkers in predicting intestinal injury, such as diamine oxidase (DAO) (14), D-lactic acid (D-LA) (15), lipopolysaccharide (LPS) (16), and (1,3)-β-D-glucan (BDG) (17).

However, there are many pathogenic mechanisms of intestinal injury in MetS patients, and there are difficulties in the evaluation of the disease status with a single biomarker. Also, there is a lack of clinical studies to assess intestinal barrier function by comparing the method of colonoscopy with serum biomarkers detection. Based on the inadequate application value of a single serum biomarker, this study aimed to combine the above serum biomarkers with other clinical features, to enhance the applied value of serum biomarkers, and to provide a new diagnostic tool for non-invasive, rapid, and accurate clinical assessment of intestinal barrier function in patients with MetS.

## Methods

### Study design

In this study, we included patients with MetS who underwent colonoscopy at the Endoscopy Center of the First Affiliated Hospital of Chongqing Medical University between March and July 2024. We collected their peripheral venous blood on the day of admission. The intestines of the participants were examined by an electronic colonoscope (Olympus CV-70). According to the results of the colonoscopy, the participants were categorized into the intestinal injury and non-intestinal injury groups. This study was endorsed by the Ethics Committee of the First Affiliated Hospital of Chongqing Medical University (No. 2024-219-01).

### Diagnostic criteria of MetS and intestinal injury

Diagnostic criteria of MetS (18): diagnosis is confirmed when three or more of the following components are present: (1) Abdominal obesity (central obesity): waist circumference ≥90 cm in men and ≥85 cm in women; (2) Hyperglycemia: fasting plasma glucose ≥6.1 mmol/L or 2-hour postprandial glucose ≥7.8 mmol/L and/or previously diagnosed diabetes under treatment; (3) Hypertension: blood pressure ≥130/85 mmHg (1 mmHg=0.133 kPa) and/or previously confirmed hypertension under treatment; (4) Fasting triglyceride (TG) ≥1.70 mmol/L; (5) Fasting high-density lipoprotein cholesterol (HDL-C) <1.04 mmol/L. The waist circumference cut-off for central obesity follows the standard established by the Health Industry Standard of the People's Republic of China-Criteria of Weight for Adults (Standard No. WS/T 428-2013) issued by the National Health and Family Planning Commission in 2013.

Diagnostic criteria of intestinal injury (19): Patients are considered to have intestinal injury if they exhibit gastrointestinal symptoms such as nausea, vomiting, abdominal pain, or diarrhea, along with imaging evidence (e.g., endoscopy, abdominal ultrasound, or computed tomography) confirming gastrointestinal bleeding, edema, ulcers, or other related findings.

### Inclusion and exclusion criteria

The inclusion criteria included: (1) patients who met the diagnostic criteria of MetS; (2) patients who were aware of the study purpose and voluntarily signed an informed consent form; (3) patients who were able to cooperate in completing bowel preparation and colonoscopy.

The exclusion criteria included: (1) pregnant women, lactating women, and those under 18 years of age; (2) patients with malignant tumors, congenital heart disease, and other serious diseases; (3) recent use of non-steroidal anti-inflammatory drugs; (4) gastrointestinal infection; (5) intestinal obstruction and other primary intestinal diseases; (6) patients with a previous history of intestinal surgery.

### Data collection

The clinical characteristics of the participants were recorded, including sex, household registration, age, body mass index (BMI), systolic blood pressure (SBP), diastolic blood pressure (DBP), history of hypertension, history of coronary artery disease, history of alcohol consumption, and history of smoking; laboratory indicators, including white blood cell (WBC), red blood cell (RBC), neutrophil percentage (NEUT%), alanine aminotransferase (ALT), fasting blood glucose (FBG), fasting serum insulin (FINS), homeostatic model assessment of insulin resistance (HOMA-IR), microalbuminuria (MAU), uric acid (UA), creatinine (Cr), total cholesterol (TC), TG, low-density lipoprotein cholesterol (LDL-C), HDL-C; serum biomarkers, including LPS, DAO, D-LA, and BDG. On the day of admission, 10 mL of peripheral venous blood was collected from the participants in a fasting state. Blood cell counts were detected by an automated hematology analyzer (Mindray Bio-Medical Electronics Co., Ltd.; Model number: BC-7500 CS), and the blood specimens were centrifuged (15 min, 1500 g), then the supernatants were tested for levels of clinical biochemical indicators by an automatic biochemistry analyzer (Beckman Coulter, Inc.; Model number: AU5821). Morning midstream clean-catch urine specimens were collected from study participants, and MAU levels were quantitatively determined using immunoturbidimetric analysis. Serum biomarkers were quantified through enzyme-linked immunosorbent assay (DAO and D-LA kits were purchased from UPing Biotechnology Technology Co., Ltd., BDG kit was purchased from Shanghai Enzyme-linked Biotechnology Co., Ltd., LPS kit was purchased from Wuhan Fine Biotech Co., Ltd.). Conduct the tests strictly in accordance with the kit instructions.

### Statistical analyses

We used SPSS version 25.0 and R version 4.4.1 software packages for statistical analyses. The data were analyzed to check for normal distribution and quantitative variables. Those that met normal distribution criteria were expressed as mean ± standard deviation and were compared using the Student’s t-test. Alternatively, the quantitative variables that were not normally distributed were presented as median (interquartile range, 25%-75%) and were compared using the Mann-Whitney U-test. Categorical variables were expressed as numbers and percentages, and the chi-square test was used for intergroup comparisons. The indicators with statistically significant differences were selected by univariate analysis, and the independent risk factors were further analyzed by multivariate logistic regression (forward logistic regression method). To develop a prediction model, we utilized the “rms” package in the R software, constructing a nomogram and assessing the repeatability of the model through bootstrapping (n = 1,000). We further validate the model using the 5-fold cross-validation method. The nomogram column graph visually displayed this model. We assessed the predictive capacity and effectiveness of the model by analyzing the receiver operating characteristic (ROC) curve, calibration curve, and decision curve analysis (DCA). To assess the predictive accuracy and discriminative power of the nomogram, we calculated the area under the ROC curve (AUC). The calibration curve was used to compare the predicted probabilities, computed from the nomogram, with the actual probabilities. Moreover, the study evaluated the practical clinical utility of the nomogram using DCA, based on the net benefit and threshold probabilities. The P value of < 0.05 indicated statistical significance.

## Results

### Participant data

**Figure 1:**
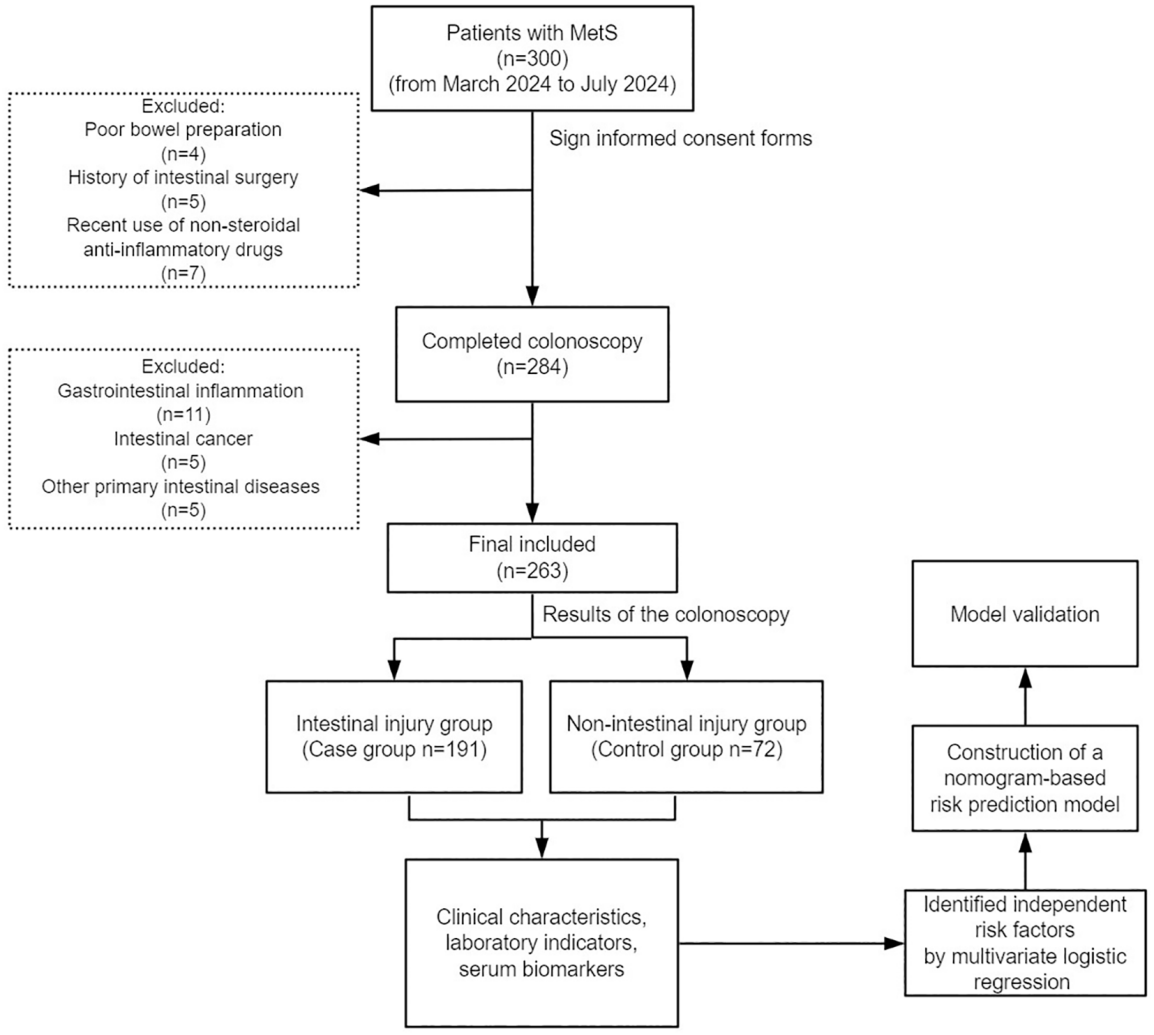
Flow diagram of study design. MetS, metabolic syndrome.

**Figure 1** shows the participant enrollment process for this study. Based on the selection criteria, 263 patients with MetS were included in this study, including 191 (72.6%) individuals with intestinal injury and 72 (27.4%) without intestinal injury.

**Table 1** displays the basic data of all participants. Compared with the non-intestinal injury group, individuals in the intestinal injury group were older [60.00 (51.00, 69.00) vs. 51.50 (39.25, 57.00)] and had a higher BMI [24.44 (22.04, 26.67) vs. 23.43 (21.64, 24.96)].

**Table 1:** Basic data of patients in the intestinal injury and non-intestinal injury groups.

Basic data	Study Participants (n = 263)		χ²/z/t	P value
	Intestinal injury group (n = 191)	Non-intestinal injury group (n = 72)		
Sex (n, %)			2.436	0.119^c^
Female	65 (34.0)	32 (44.4)		
Male	126 (66.0)	40 (55.6)		
Household registration (n, %)			1.260	0.262^c^
Rural	73 (38.2)	33 (45.8)		
Urban	118 (61.8)	39 (54.2)		
Age (years)	60.00 (51.00, 69.00)	51.50 (39.25, 57.00)	-5.335	<0.001^a^
BMI (kg/m^2^)	24.44 (22.04, 26.67)	23.43 (21.64, 24.96)	-2.507	0.012^a^
History of hypertension (n, %)			0.072	0.789^c^
Yes	151 (79.1)	58 (80.6)		
No	40 (20.9)	14 (19.4)		
SBP (mmHg)	143.73 (129.25, 155.31)	142.97 (133.38, 148.62)	-0.851	0.395^a^
DBP (mmHg)	82.31 (76.47, 89.36)	80.37 (76.65, 88.45)	-0.355	0.723^a^
History of coronary artery disease (n, %)			1.240	0.265^c^
Yes	113 (59.2)	48 (66.7)		
No	78 (40.8)	24 (33.3)		
History of alcohol consumption (n, %)			4.619	0.032^c^
Yes	66 (34.6)	15 (20.8)		
No	125 (65.4)	57 (79.2)		
History of smoking (n, %)			0.727	0.394^c^
Yes	61 (31.9)	27 (37.5)		
No	130 (68.1)	45 (62.5)		
WBC (10^9^/L)	7.55 (5.80, 9.41)	7.51 (5.60, 9.41)	-0.205	0.838^a^
RBC (10^12^/L)	4.24 ± 0.87	4.25 ± 0.85	0.073	0.942^b^
NEUT%	85.20 (78.50, 88.90)	70.65 (61.65, 79.70)	-7.850	<0.001^a^
ALT (U/L)	32.00 (25.90, 36.10)	32.45 (26.30, 35.15)	-0.109	0.913^a^
FBG (mmol/L)	5.72 (4.58, 6.38)	5.48 (4.56, 6.37)	-0.517	0.605^a^
FINS (mIU/L)	12.71 (10.35, 14.32)	12.30 (10.33, 13.88)	-1.164	0.245^a^
HOMA-IR	3.08 (2.49, 3.74)	3.03 (2.46, 3.53)	-0.990	0.322^a^
MAU (mg/L)	178.04 (103.45, 204.84)	154.37 (89.84, 201.34)	-1.869	0.062^a^
UA (μmol/L)	340.26 (294.38, 403.27)	321.40 (287.25, 399.04)	-0.899	0.369^a^
Cr (μmol/L)	87.30 (68.60, 104.00)	83.40 (67.80, 96.78)	-1.320	0.187^a^
TC (mmol/L)	5.99 (4.67, 6.62)	5.67 (4.54, 6.43)	-0.903	0.367^a^
TG (mmol/L)	1.81 (1.33, 2.40)	2.10 (1.58, 2.79)	-2.185	0.029^a^
LDL-C (mmol/L)	3.56 (2.94, 4.21)	3.32 (3.04, 4.17)	-0.381	0.703^a^
HDL-C (mmol/L)	0.94 (0.79, 1.02)	1.00 (0.82, 1.15)	-1.819	0.069^a^
LPS (ng/mL)	12.37 (11.02, 19.38)	9.60 (4.43, 12.35)	-6.420	<0.001^a^
DAO (ng/mL)	15.13 (12.75, 17.90)	11.69 (9.37, 12.78)	-8.312	<0.001^a^
D-LA (μmol/L)	21.26 (19.25, 25.19)	21.10 (18.98, 23.25)	-1.168	0.243^a^
BDG (pg/mL)	73.26 (45.26, 90.48)	67.69 (35.66, 90.47)	-0.891	0.373^a^

ALT, alanine aminotransferase; BDG, (1,3)-β-D-glucan; BMI, body mass index; Cr, creatinine; DAO, diamine oxidase; DBP, diastolic blood pressure; D-LA, D-lactic acid; FBG, fasting blood glucose; FINS, fasting serum insulin; HDL-C, high-density lipoprotein cholesterol; HOMA-IR, homeostatic model assessment of insulin resistance; LDL-C, low-density lipoprotein cholesterol; LPS, lipopolysaccharide; MAU, microalbuminuria; NEUT%, neutrophil ratio; RBC, red blood cell; SBP, systolic blood pressure; TC, total cholesterol; TG, triglyceride; UA, uric acid; WBC, white blood cell. ^a^Results shown as median and interquartile range and analyzed using Mann-Whitney U-test. ^b^Results shown as mean ± standard and analyzed using Student’s t-test. ^c^Chi-square test was used for proportions comparison.

### Univariate analysis

The results of the univariate analysis are shown in **Table 2**. There were significant differences in age (P < 0.001), history of alcohol consumption (P = 0.034), BMI (P = 0.012), NEUT% (P < 0.001), LPS (P < 0.001) and DAO (P < 0.001) levels between the two groups (P < 0.05).

**Table 2:** Univariate and multivariate logistic regression analysis of independent risk factors for intestinal injury in patients with MetS.

Variables	Univariate analysis		Multivariate analysis	
	P value	OR (95% CI)	P value	OR (95% CI)
Sex	0.120	1.551 (0.892-2.696)	–	–
Household registration	0.263	1.368 (0.791-2.365)	–	–
Age	**<0.001**	1.054 (1.032-1.077)	**<0.001**	1.071 (1.033-1.111)
BMI	**0.012**	1.118 (1.025-1.220)	**0.029**	1.229 (1.021-1.478)
History of hypertension	0.789	0.911 (0.462-1.798)	–	–
SBP	0.200	1.011 (0.994-1.028)	–	–
DBP	0.791	1.005 (0.970-1.041)	–	–
History of coronary artery disease	0.266	0.724 (0.410-1.279)	–	–
History of alcohol consumption	**0.034**	2.006 (1.056-3.813)	0.177	2.046 (0.724-5.779)
History of smoking	0.394	0.782 (0.444-1.377)	–	–
WBC	0.488	1.031 (0.945-1.125)	–	–
RBC	0.941	0.988 (0.721-1.355)	–	–
NEUT%	**<0.001**	1.105 (1.074-1.138)	**<0.001**	1.116 (1.067-1.166)
ALT	0.850	1.004 (0.966-1.042)	–	–
FBG	0.582	1.073 (0.835-1.379)	–	–
FINS	0.072	1.089 (0.993-1.194)	–	–
HOMA-IR	0.115	1.277 (0.942-1.730)	–	–
MAU	0.090	0.996 (0.992-1.001)	–	–
UA	0.334	1.002 (0.998-1.005)	–	–
Cr	0.148	1.010 (0.996-1.024)	–	–
TC	0.399	1.101 (0.881-1.375)	–	–
TG	0.877	0.990 (0.873-1.123)	–	–
LDL-C	0.946	1.011 (0.734-1.392)	–	–
HDL-C	0.108	0.407 (0.135-1.220)	–	–
LPS	**<0.001**	1.266 (1.170-1.370)	**<0.001**	1.308 (1.171-1.461)
DAO	**<0.001**	1.489 (1.328-1.669)	**<0.001**	1.736 (1.426-2.114)
D-LA	0.143	1.050 (0.984-1.121)	–	–
BDG	0.334	1.004 (0.995-1.013)	–	–

The variables with univariate regression P value of < 0.05 were included in multivariate regression analysis. Bolded P value indicates statistical significance.

ALT, alanine aminotransferase; BDG, (1,3)-β-D-glucan; BMI, body mass index; Cr, creatinine; DAO, diamine oxidase; DBP, diastolic blood pressure; D-LA, D-lactic acid; FBG, fasting blood glucose; FINS, fasting serum insulin; HDL-C, high-density lipoprotein cholesterol; HOMA-IR, homeostatic model assessment of insulin resistance; LDL-C, low-density lipoprotein cholesterol; LPS, lipopolysaccharide; MAU, microalbuminuria; NEUT%, neutrophil ratio; RBC, red blood cell; SBP, systolic blood pressure; TC, total cholesterol; TG, triglyceride; UA, uric acid; WBC, white blood cell.

### Multivariate analysis

Indicators with statistical differences in the above univariate analysis were further analyzed by multivariate binary logistic regression (forward logistic regression method) to identify independent risk factors. Based on the results of the multivariate analysis (**Table 2**), age (Odds Ration [OR] = 1.071, 95% Confidence Interval [CI] = 1.033-1.111, P < 0.001), BMI (OR = 1.229, 95% CI = 1.021-1.478, P = 0.029), NEUT % (OR = 1.116, 95% CI = 1.067-1.166, P < 0.001), LPS (OR = 1.308, 95%CI = 1.171-1.461, P < 0.001), and DAO (OR = 1.736, 95%CI = 1.426-2.114, P < 0.001) were observed to be independent risk factors for intestinal injury in patients with MetS.

### Construction of a nomogram-based risk prediction model

**Figure 2:**
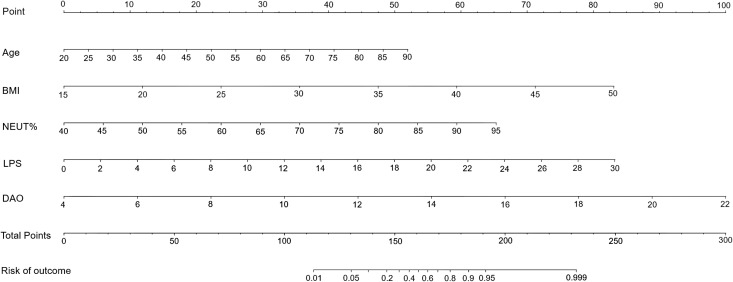
A nomogram predicting the risk of intestinal injury in patients with MetS. BMI, body mass index; DAO, diamine oxidase; LPS, lipopolysaccharide; MetS, metabolic syndrome; NEUT%, neutrophil percentage.

Using the five independent risk factors identified through multivariate logistic regression analyses, we designed a nomogram to predict the risk of intestinal injury in patients with MetS. The nomogram presented in **Figure 2** illustrates a model that employs points, predictors, total points, and the risk of occurrence. The scale of each line segment signifies the range of values for the corresponding predictor, with the length representing the contribution of the predictor to the risk of intestinal injury. The top point in the figure corresponds to the points of each predictor for varying values. The tick marks along each variable’s axis represent its value range. Using a straightedge, clinicians can get individual scores of each risk factor in nomogram, then summed these scores to obtain a total score. This total score is projected downward to align with the risk probability axis, enabling rapid calculation of intestinal injury risk for each patient with MetS. The higher the total points, the greater the intestinal injury risk for the patient. Using 0.783 as the critical threshold, patients with risk values exceeding this level are considered at risk for intestinal injury and require enhanced early intervention and health management.

### Model performance

**Figure 3:**
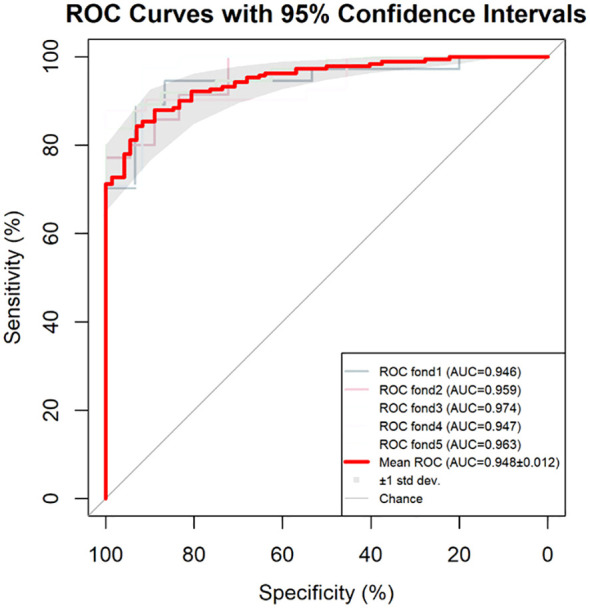
Results of the 5-fold cross-validation for the model. AUC, area under the curve; ROC, receiver operating characteristic.

The repeatability of the model was confirmed through the bootstrap method, which involved resampling 1,000 times. The accuracy was 0.873, and the Kappa value was 0.672, demonstrating that the repeatability of the model was satisfactory. The results of the 5-fold cross-validation are shown in **Figure 3**. All the AUC values were ≥0.946, and the AUC of the average ROC curve was 0.948 ± 0.012. The results indicate that the prediction effectiveness of this model is good.

**Figure 4:**
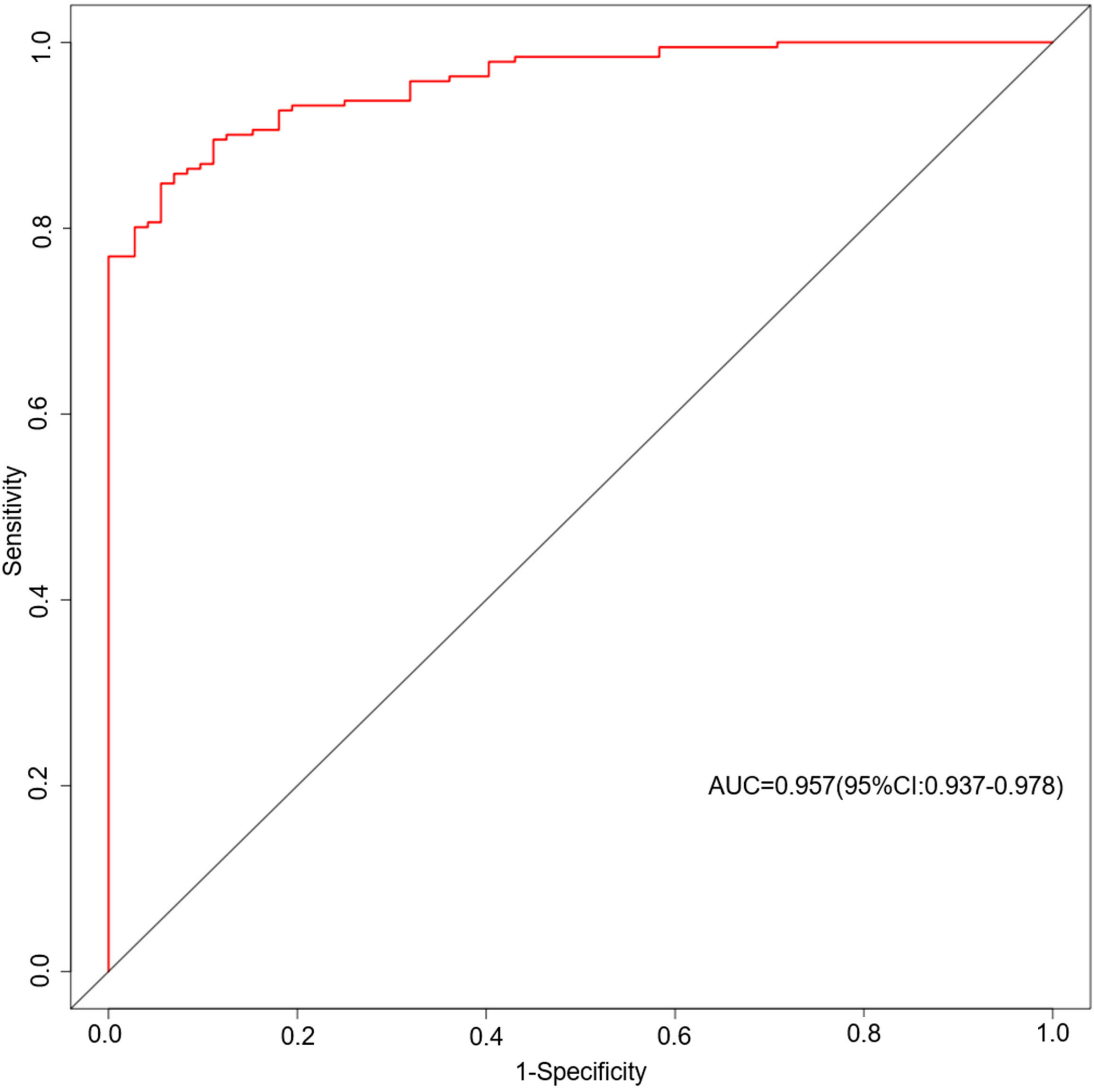
ROC curves based on the nomogram model predicting the risk of intestinal injury in patients with MetS. AUC, area under the curve; MetS, metabolic syndrome; ROC, receiver operating characteristic.

**Figure 5:**
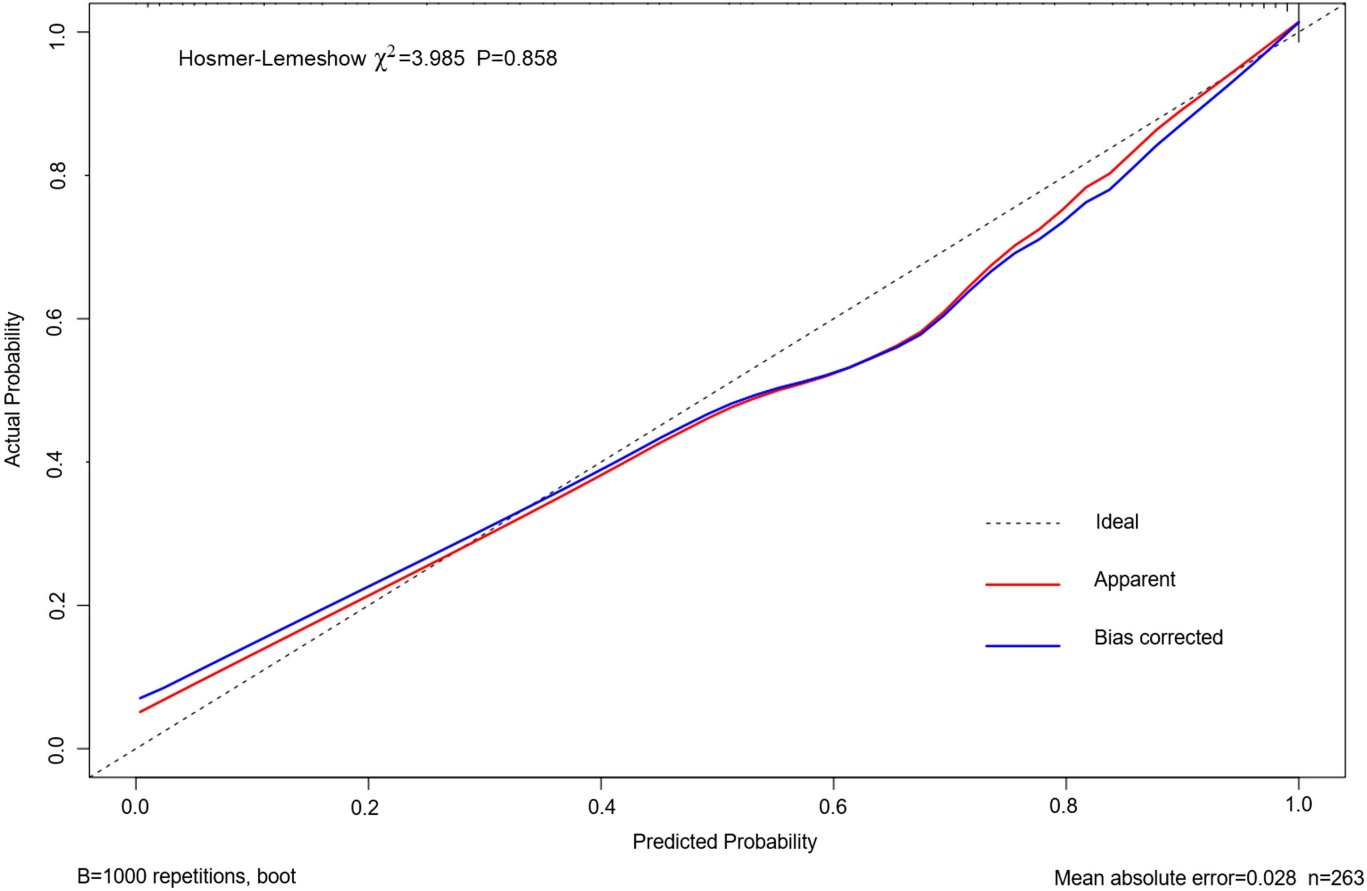
Calibration curve based on the nomogram model predicting the risk of intestinal injury in patients with MetS. MetS, metabolic syndrome.

**Figure 6:**
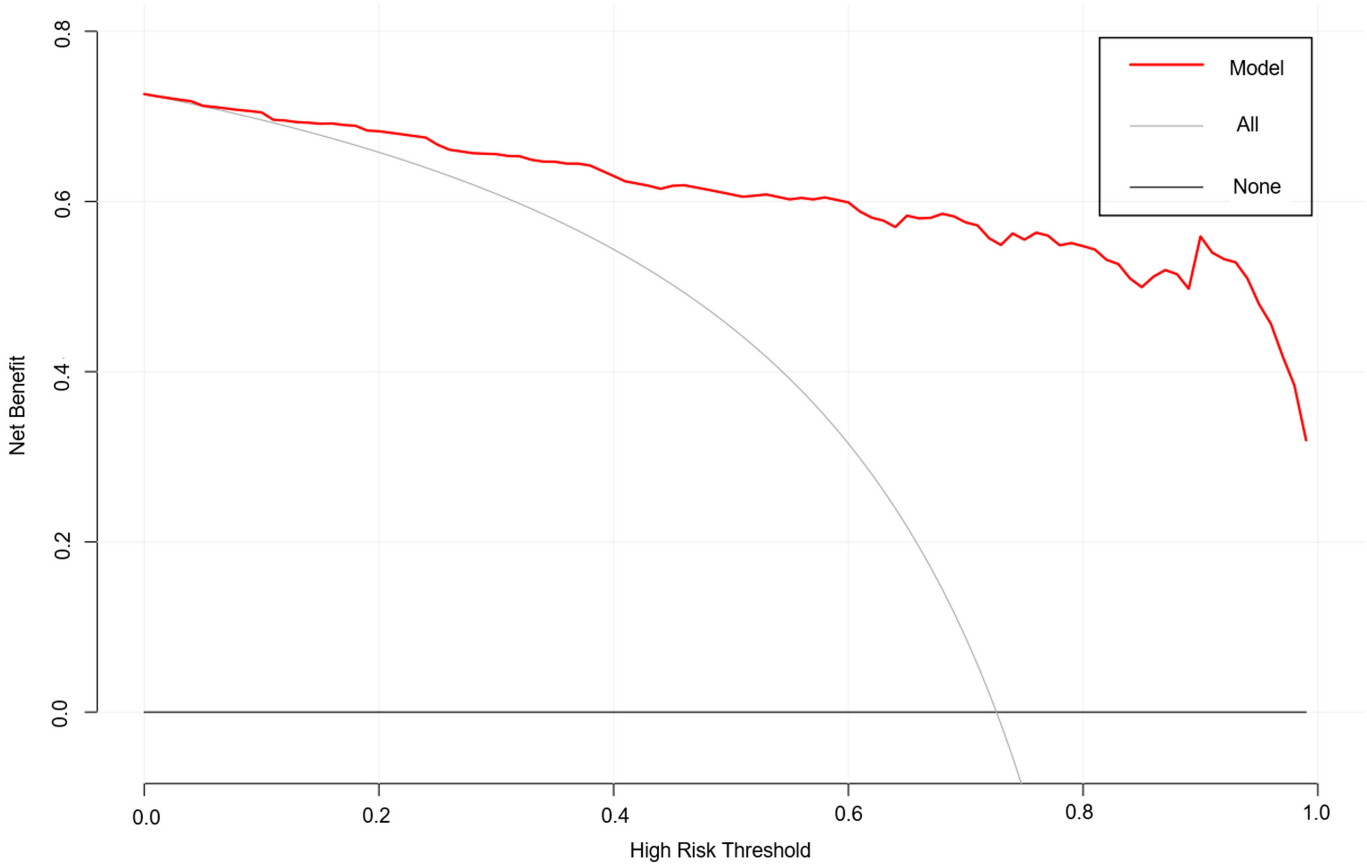
DCA results based on the nomogram model predicting the risk of intestinal injury in patients with MetS. DCA, decision curve analysis; MetS, metabolic syndrome.

**Figure 7:**
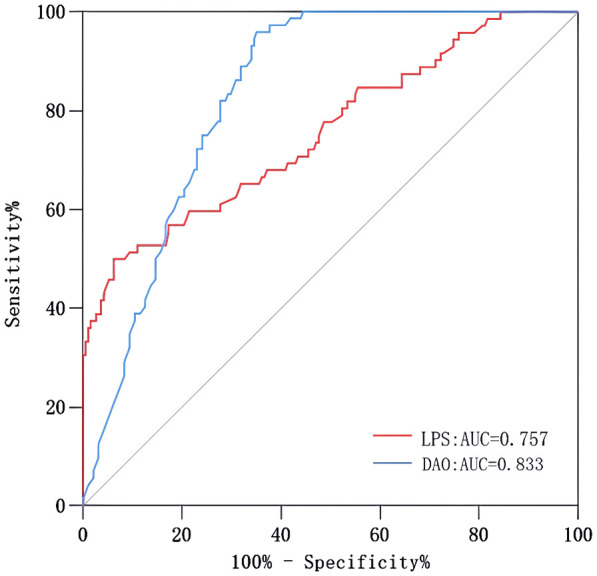
ROC curves of LPS and DAO. AUC, area under the curve; DAO, diamine oxidase; LPS, lipopolysaccharide; MetS, metabolic syndrome; ROC, receiver operating characteristic.

As shown in **Figure 4**, the area under the ROC curve was 0.957 (95% CI: 0.937-0.978), the maximum Youden index was 0.793, the optimal cut-off value was 0.783, the sensitivity was 0.848, and the specificity was 0.944. Our findings demonstrated that the model exhibited strong predictive accuracy and differentiation. **Figure 5** displays the calibration curve of the model, indicating that the predicted probability of the model is in good agreement with the actual observed probability. The Hosmer-Lemeshow test results showed a robust goodness of fit (χ^2^ = 3.985, P = 0.858). **Figure 6** displays the results of DCA for this nomogram-based risk prediction model. Based on the results of the DCA, using this model to predict intestinal injury in patients with MetS would add more benefit when the threshold probability was > 7%, compared with employing either treat-all or treat-none strategies. **Figure 7** shows the predictive value of LPS and DAO regarding intestinal injury risk in patients with MetS. The AUC of LPS was 0.757 (95% CI: 0.687-0.826), the maximum Youden index was 0.437, the optimal cut-off value was 9.410 ng/mL, the sensitivity was 0.937, and the specificity was 0.500. The AUC of DAO was 0.833 (95% CI: 0.785-0.879), the maximum Youden index was 0.608, the optimal cut-off value was 13.764 ng/mL, the sensitivity was 0.649, and the specificity was 0.958. Through analysis, it can be seen that the AUCs of these two indicators are both greater than 0.7, indicating that the prediction has a high degree of accuracy. This also demonstrates that the combined use of multiple biomarkers yields a higher AUC compared to using a single serum biomarker.

## Discussion

### Factors associated with intestinal injury in patients with MetS

The present report was a hospital-based case-control study comprising patients with MetS who underwent colonoscopy in the Endoscopy Center of our hospital. Our findings indicated that age, BMI, NEUT%, DAO, and LPS values were elevated in the intestinal injury group compared with the non-intestinal injury group, and our binary logistic regression model analysis revealed that the above parameters were risk factors for the occurrence of intestinal injury in patients with MetS.

Our current study demonstrated that age was an independent risk factor for intestinal injury in patients with MetS, which was consistent with previous studies (20). Based on these statistics, the occurrence of MetS among the elderly in China is 36.9% (21). With age, the changes in the composition of the microbiome will produce an inflammatory environment in the intestine, leading to bacterial components entering the systemic circulation and triggering inflammation. This suggests that we should pay more attention to gut health in older patients with MetS.

Several studies have highlighted that obesity contributes to various chronic diseases and increases intestinal permeability (22, 23). The obesity-related intestinal flora alters host intestinal homeostasis, further triggering inflammation. BMI was an independent risk factor for intestinal injury in patients with MetS. For each one-unit increase in BMI, the probability of developing intestinal injury in patients with MetS increased by 22.9%. A study (24) showed that elderly people aged 60 to 74 years should keep their BMI below 24.0 kg/m^2^, while those aged 75 years and above should aim for a BMI value below 23.0 kg/m^2^. This may help reduce the risk of chronic metabolic diseases.

We found that NEUT% can act as a predictor of intestinal injury in patients with MetS, possibly due to neutrophil involvement in inflammation. Some studies have shown that neutrophils have a dual function in the progression of inflammatory bowel disease, clearing intestinal microorganisms to maintain the functional stability of intestinal mucosa, whereas an excessive inflammatory response will lead to further damage to the body (25). Neutrophils are significantly associated with MetS and their levels are proportional to its severity (26). In addition, several studies have used the neutrophil-lymphocyte ratio to evaluate acute and chronic inflammation in MetS (27, 28). Patients with MetS often have diabetes mellitus, and their immune function is affected, so neutrophil counts in some patients may be normal or only slightly increased. Therefore, NEUT% is more sensitive to changes in blood inflammatory cell counts.

Hypertension is a prevalent component of MetS and exhibits close associations with both insulin resistance and dyslipidemia (29). Among all participants in this study, 79.5% were diagnosed with hypertension. The hypertension prevalence was 79.1% in the intestinal injury group and 80.6% in the non-intestinal injury group, with no statistically significant difference between the two groups. Regarding SBP and DBP, the intestinal injury group showed higher levels compared to the non-intestinal injury group, though the differences were not statistically significant. Hypertension can lead to microstructural changes in the intestine, thereby impairing intestinal function. Furthermore, hypertensive patients often exhibit gut microbiota dysbiosis, which promotes toxin translocation into the systemic circulation, triggering a systemic inflammatory response (30, 31). Therefore, controlling blood pressure is crucial for stabilizing the condition of patients with MetS.

The core mechanism underlying MetS is insulin resistance. Once insulin resistance develops, the body’s sensitivity and responsiveness to the physiological effects of insulin are reduced (32, 33). Recent studies have revealed that insulin resistance is present at all stages of MetS and is closely associated with cardiovascular disease risk. An animal study revealed that insulin resistance induces early and reversible dysbiosis-mediated intestinal barrier damage and dysfunction (34). Our findings revealed that among all participants, 243 (92.4%) exhibited insulin resistance. In the intestinal injury group, 11 (5.8%) did not show insulin resistance, whereas in the non-intestinal injury group, 9 (12.5%) were without insulin resistance. Moreover, the incidence and severity of insulin resistance increased with the number of MetS components. Compared to the non-intestinal injury group, the intestinal injury group had higher FINS and HOMA-IR levels. However, there were no statistically significant differences in FINS and HOMA-IR between the two groups. This indicates that although insulin resistance is the core mechanism underlying the onset of MetS, it is not an effective indicator for evaluating intestinal injury in patients with MetS.

The serum DAO levels were higher in the intestinal injury group, and this is consistent with studies in animal histopathology (35). DAO is a biomarker of intestinal mucosal cells that occurs in the upper layer of the villi of the mammalian small intestine and is almost absent in other tissues and cells. Under physiological conditions, the activity of DAO in plasma is minimal. However, when the gut mucosa is impaired, the concentration of DAO in the blood rises dramatically due to the release from intestinal mucosal cells. So, it is a good indicator of the structural integrity of the intestinal mucosa (36). Our findings also show that the higher the serum LPS levels, the greater the probability of intestinal injury. LPS originates from intestinal bacteria, and it is difficult for it to enter the blood circulation due to an intact intestinal barrier in healthy people (37). When the intestinal barrier is impaired or mucosal permeability increases, LPS enters the blood circulation through epithelial translocation, giving rise to endotoxemia. Hence, LPS is an indicator for evaluating bacterial translocation. These results suggest that DAO and LPS levels may be used as predictors for intestinal injury in patients with MetS.

MAU is a sensitive diagnostic marker of early renal injury. Beyond intrinsic renal pathology, elevated MAU correlates strongly with cardiovascular risk and may represent an independent predictor of cardiovascular events (38). In this study, the MAU level in the intestinal injury group was higher than that in the non-intestinal injury group. Some studies have found that the more components of MetS present, the higher the incidence of MAU (39–41). However, no direct studies currently exist regarding the relationship between intestinal injury and MAU. Univariate analysis revealed no statistically significant difference in MAU levels between the two groups, indicating that MAU is not an effective indicator for assessing intestinal injury in patients with MetS. It should be noted that MAU test results may be influenced by various factors.

D-LA is a metabolite of bacterial fermentation, and mammals lack the enzyme system to rapidly degrade it. When intestinal mucosal permeability increases, large amounts of D-LA enter the bloodstream through the damaged mucosa (42). Therefore, serum D-LA levels can reflect the extent of intestinal mucosal damage. BDG is widely distributed in the cell walls of various fungi. When the fungus enters the bloodstream or deep tissues of the human body, BDG is released from the fungal cell wall (43). The serum BDG levels are significant for intestinal fungal infection. Our present data showed that serum D-LA and BDG levels in the intestinal injury group were higher than those in the non-intestinal injury group. Nevertheless, there was no statistically significant difference in D-LA and BDG levels among the two groups, which was inconsistent with some previous studies that used them as markers of intestinal damage. We speculate that these anomalous results may be due to the following reasons: some studies have suggested that both obesity and the use of metformin are known factors associated with high levels of lactic acid (44), and lactic acid is closely linked to type 2 diabetes mellitus (45). Due to the complexity of MetS, the pathophysiological changes in its various disease components have an impact on lactic acid metabolism. Previous studies have targeted only one disease component of MetS and cannot be fully applied to patients with MetS. On the other hand, patients with diabetes, especially those with acute complications, are prone to various bacterial or even fungal infections (46). To control the inflammation post-infection, it is often necessary to use a variety of broad-spectrum antibiotics for a long time, thus making deep fungal infections worse. As a result, serum BDG levels cannot accurately reflect the extent of intestinal damage in patients with MetS. Currently, there is little research on biomarkers of intestinal damage in patients with MetS, while the impact of MetS on the gut cannot be ignored. As the number of patients with MetS continues to grow, there is a greater need for research in this area.

### The scientific validity of the nomogram prediction model for intestinal injury risk in patients with MetS

In recent years, some risk prediction models have been established and applied to various diseases. Ukah et al. (47) developed a prediction model for type 2 diabetes mellitus complications in women with gestational diabetes mellitus (GDM). This model demonstrated good discrimination [AUC: 0.72 (95% CI 0.69-0.74)] and satisfactory calibration (slope≥0.9). With moderate predictive accuracy, this model may prove to be a clinically useful tool for post-GDM management after external validation. Yuan et al. (48) developed a nomogram model for risk management before the treatment of constipation in patients with type 2 diabetes mellitus, using variables such as age, glycated hemoglobin, blood calcium concentration, anxiety, and exercise status. This prediction model exhibits good performance and clinical application value. Li et al. (49) constructed a risk prediction model, which guides the clinical prevention and control of gastrointestinal motility disorders in the diabetic population. As far as we know, there is no risk prediction model for intestinal injury targeting the population with MetS. Given the continuous growth trend of the MetS population in the world today and the “invasive” characteristics of colonoscopy, it is necessary to construct an accurate and efficient prediction model for the MetS population. The model used in this study to predict the risk of intestinal injury is based on serum biomarkers and clinical characteristics. Generally, for a predictive model, an AUC value of the ROC curve between 0.7 and 0.9 indicates good predictive power. Below this value, the predictive power is low, and above this value, the predictive power is strong. The AUC value in this study was 0.957, indicating the strong predictive power of the model. Based on the ROC curve analysis of the predictive model, the cut-off value corresponding to the maximum Youden’s index (0.793) was identified as the optimal cut-off value (0.783) for assessing intestinal injury risk in patients with MetS. Using 0.783 as the critical threshold, patients with risk values exceeding this level are considered at risk for intestinal injury and suggests the necessity of early intervention and health management. The higher the total score obtained by the model, the greater the risk of intestinal injury. It can be used as an auxiliary diagnostic pathway to effectively avoid the limitations of colonoscopy. Compared with traditional colonoscopy, this model is non-invasive and can be used when colonoscopy is unavailable or the patient does not qualify for colonoscopy. Thus, our nomogram-based risk prediction model can clinically benefit patients with MetS in evaluating intestinal injury. We recommend that this model be widely used in primary hospitals to benefit more patients with MetS.

## Conclusion

The risk prediction model developed in this study consisted of five predictors, including age, BMI, NEUT%, DAO, and LPS. Our model had an excellent overall performance after bootstrapping, the 5-fold cross-validation, ROC curve analysis, calibration curve input, and DCA. Further, this model can help healthcare professionals simply and accurately predict the risk of intestinal injury in patients with MetS, which may bring significant benefits to these patients. However, our study has some limitations. This study lacks a validation cohort, which may lead to unreliable performance evaluation of the model. It is a single-center study with a small sample size under the constraints of external factors, thus, the conclusions drawn may have a certain degree of deviation. The clinical significance of this prediction model should be further verified with external data and evaluated in larger cohorts in the future. It is necessary to cooperate with other medical institutions or research teams to conduct multi-center studies and expand the sample size to further improve the reliability and statistical efficacy of the experimental results.

## Data Availability

The original contributions presented in the study are included in the article/supplementary material. Further inquiries can be directed to the corresponding authors.
